# Satisfaction and Public Health Cost of a Statewide Influenza Nurse Triage Line in Response to Pandemic H1N1 Influenza

**DOI:** 10.1371/journal.pone.0050492

**Published:** 2013-01-15

**Authors:** Alicen B. Spaulding, Deborah Radi, Heather Macleod, Ruth Lynfield, Michelle Larson, Terri Hyduke, Peter Dehnel, Aaron S. DeVries

**Affiliations:** 1 School of Public Health, University of Minnesota, Minneapolis, Minnesota, United States of America; 2 Minnesota Department of Health, St. Paul, Minnesota, United States of America; 3 Children’s Physician Network, Minneapolis, Minnesota, United States of America; University of North Carolina School of Medicine, United States of America

## Abstract

**Background:**

The 2009 H1N1 pandemic strained healthcare systems. There was a need for supportive services, rapid antiviral access, and minimization of unnecessary healthcare contacts particularly face-to-face interactions. In response, the Minnesota Department of Health (MDH) launched a telephone-based nurse triage line (NTL) called the Minnesota FluLine coordinating all major MN healthcare systems with NTLs to form a single toll-free number triage service. Callers were evaluated for symptoms of influenza-like illness (ILI) and were prescribed an antiviral if indicated, using nurse administered protocols.

**Methods:**

To determine caller outcomes, associated healthcare seeking, and satisfaction a telephone survey of Minnesota FluLine callers was conducted using a 5% random sample of those who completed the protocol and those who did not.

**Results:**

Of 6,122 callers with ILI who began the nurse protocol administered by the contract NTL, 1,221 people were contacted for the survey and 325 agreed to participate; response rate was 26%. Of those who completed the nurse protocol 73% said they would have sought healthcare without the Minnesota FluLine, 89% reported the service was moderately or very helpful, and 91% reported being satisfied or very satisfied. Of those not completing the protocol, 50% reported the service was moderately or very helpful and 50% reported being satisfied or very satisfied. 72% of qualitative responses to open-ended questions were positive regarding the MN FluLine. Cost to MDH for operating the Minnesota FluLine service was $331,226 to service 27,391 callers ($12.09/call).

**Discussion:**

The Minnesota FluLine diverted patients with mild ILI symptoms away from acute care visits at low cost and had a high rate of satisfaction among callers. Early intervention likely prevented morbidity and possibly additional cases. NTLs are powerful and flexible tools for pandemic response and should be considered as an important tool for future emergency responses.

## Introduction

The pandemic caused by 2009 H1N1 had a substantial impact leading to an estimated 43 to 89 million cases and 8,870 to 18,300 deaths in the United States from April 2009 to April 2010 [1]. Because antivirals are most effective if given early in the course of infection, there was a need to quickly evaluate patients, particularly those with underlying health conditions at risk for more severe disease. In addition, there were concerns about spread of influenza in slow-moving, overcrowded waiting rooms. Many healthcare facilities struggled to respond to the increased number of patients with symptoms of influenza and to those who sought care out of concern for influenza but did not have symptoms (worried well). In the state of Minnesota, influenza activity peaked first in June 2009, abated during July and August, and increased in activity once again in September 2009 coinciding with the return of students to school [2], [3]. As healthcare utilization began to increase, based on reports to Minnesota Department of Health (MDH) from hospitals, clinics, and nurse triage telephone service providers, there were gaps in meeting the specific needs of those with influenza-like illness (ILI; defined as fever >100°F (37.8°C) with cough and/or sore throat) or exposure to someone with ILI, in particular those who were uninsured or underinsured.

To meet these needs, the MDH and Minnesota healthcare systems (defined as healthcare organizations that provide healthcare insurance coverage as well as healthcare delivery organizations) sought a mechanism to support the existing healthcare delivery system, improve access to antivirals if needed, and minimize unnecessary healthcare interactions. Telephone-based evaluation and triage have been utilized in many areas of medicine including management of chronic disease and acute illnesses with similar outcomes compared to in-person care [4]–[7]. A telephone-based nurse triage line (NTL) service was designed and implemented by MDH in collaboration with all Minnesota-based healthcare systems with existing NTLs. This service was called the MN FluLine and began service on October 21, 2009.

Other NTLs were utilized during the response to 2009 H1N1 [8]–[12]. We have previously described how the MN FluLine reached over 27,391 callers preventing up to an estimated 10,998 in-person healthcare contacts [13]. However to date there has not been an assessment of how NTLs were perceived by the callers nor an assessment of the costs associated with such programs. In order to evaluate this intervention, a program evaluation of the MN FluLine was conducted to further assess who utilized the service, determine caller reported outcomes, caller levels of satisfaction, and estimate costs associated with this type of intervention.

## Methods

### Ethics Statement

This project was determined to be program evaluation by the MDH Institutional Review Board (IRB) staff and did not require IRB review.

### MN FluLine Design

Specific details of the MN FluLine intervention have been previously presented [13]. In brief, all healthcare plans (organizations that provide healthcare insurance coverage) and large healthcare delivery organizations (organizations that directly provide healthcare using clinics or hospitals) with NTLs serving Minnesota collaborated to form a unified triage system with a single toll-free number serving the MN population (5.3 million) and agreed to operate in a coordinated fashion. This group of organizations represented our partner NTLs. A single NTL was selected to operate the toll-free number (the contract NTL) and serve callers who were not associated with one of the partner NTLs. A single triage protocol was created based on the CDC 2009 H1N1 influenza treatment guidelines; this was edited and approved by partner and contract NTL medical directors and was utilized by all NTLs. Callers with healthcare insurance coverage by one of the partner NTLs were transferred to their own NTL; all others were managed by the contract NTL.

Callers first spoke to a medical screener and were asked if they were sick or were calling about someone else who was sick. If the caller answered yes, contact information was collected and a registered nurse (RN) subsequently returned the call or attempted to return a call up to six times. Once contacted, callers were advised to home care, to call their doctor, to go to an emergency department (ED), or call 911 based on their symptoms using a nurse administered protocol. If callers were advised to home care and had risk factors associated with increased severity of influenza based on CDC guidelines, they were prescribed an antiviral per protocol. The nurse contacted a pharmacy of the callers’ choice and prescribed oseltamivir per protocol. Minnesota state antiviral supplies were distributed to pharmacies throughout Minnesota and were accessed if the patient was underinsured, uninsured, or there was a local disruption of oseltamivir supply [13]. Costs were assessed from the perspective of public health implementation and incurred cost by MDH for operating the MN FluLine were calculated based on call numbers.

### Caller Survey Design

A survey of callers who were contacted by an RN at the contract NTL was conducted including those who completed the nurse protocol and those who began the nurse protocol but did not complete it. Caller-specific data from the other partner NTL’s were unavailable and therefore were not included in the survey. Both surveys consisted of 8 questions with a targeted survey completion time of less than 5 minutes. Additional opportunities for open ended feedback were also included (Table 1). A modified survey was created for participants that did not complete the protocol which included individuals who ended the call prior to completion of the full triage protocol and those who could not be reached by nurses calling back. Survey calls were conducted on various days of the week and at various times throughout the day, including evenings and weekends, and at least two attempts were made per caller.

**Table 1:** List of evaluation survey questions for both evaluation survey groups.

Completed nurse protocol	Did not complete nurse protocol
How did you find out about the FluLine?	How did you find out about the FluLine?
Were you able to follow the nurse’s recommendation?	Did a nurse attempt to call you back?
If not able to follow the nurse’s recommendation, why not?	If yes, do you remember why you weren’t able to answer the call?
Did you visit a doctor, nurse, or other health professional for this illness?	Did you visit a doctor, nurse, or other health professional for this illness?
Did you receive Tamilflu* for this illness? Were you able to take it asrecommended?	Did you receive Tamilflu* for this illness? Were you able to take it as recommended?
If the MN FluLine had not been available, what would you have done?	If the MN FluLine had not been available, what would you have done?
How satisfied were you with your FluLine experience – very satisfied, satisfied, dissatisfied or very dissatisfied?	How satisfied were you with your FluLine experience – very satisfied, satisfied, dissatisfied or very dissatisfied?
How helpful was the MN FluLine to you? – very helpful, moderately helpful,slightly helpful or not at all helpful?	How helpful was the MN FluLine to you? – very helpful, moderately helpful, slightly helpful or not at all helpful?
Is there anything else you would like to tell us about your experience with the FluLine?	Is there anything else you would like to tell us about your experience with the FluLine?

* Trade name for oseltamivir. Trade name was chosen as this was likely more recognizable to survey participants.

Two 5% random samples of unique callers were selected for the telephone survey from all callers beginning the protocol administered by the contract NTL; one 5% sample among those who completed the protocol and one 5% sample among those who did not. All eligible callers were assigned a random number using a random number generator and callers were contacted in numeric order until a 5% sample was obtained. Eligible callers were those calling the MN FluLine from October 21, 2009 - February 28, 2010 and the caller survey was conducted April 1- June 4, 2010 among eligible callers. Callers from March were excluded as data were incomplete at the time of randomization and 2009 H1N1 influenza activity was diminished based on epidemiologic surveillance for hospitalized cases in Minnesota [3]. All callers were read a statement describing the purpose of the survey and that it was voluntary; the survey was then administered to those who consented.

The caller survey was administered by the same contract NTL that administered the MN FluLine. Staff with experience in conducting telephone surveys received in-person training and written instructions specific to this survey. Survey questions were piloted on 21 callers to assess comprehensibility and length of the survey; no significant changes were made prior to the final evaluation survey based on the results of the pilot. Findings from the pilot test survey were excluded from the results presented but were consistent with those obtained from the final study sample.

### Data Analysis

Data entry was conducted by a single person. Accuracy of data entry was audited by a review of a 10% random sample of data by a second person. Data analysis was conducted using Microsoft Access 2003 (Microsoft, Inc.) and SAS 9.2 (SAS Institute, Inc., Cary, NC). Differences were tested for using Student’s *t*-test, chi-square test and Fisher’s exact test where appropriate and using a significance level of p<0.05. Intervention fidelity was assumed to be 100% as we assumed all callers had the same standardized protocol administered. Qualitative feedback provided by callers was categorized as positive, negative, or neutral.

## Results

**Figure 1:**
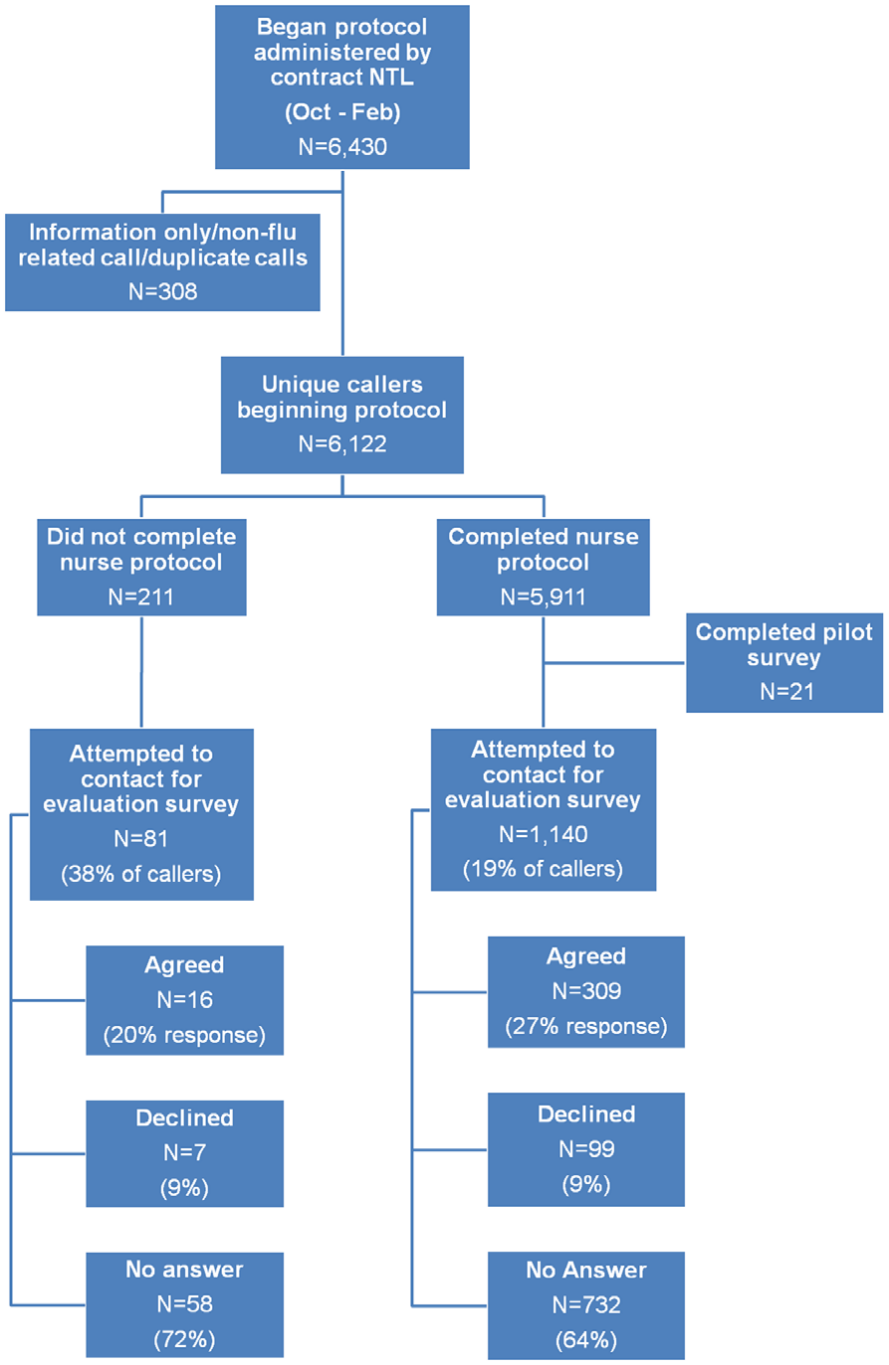
MN FluLine callers to the contract NTL from October 2009 through February 2010 who began the nurse protocol and those who participated in the evaluation survey. Callers during the month of March (n = 38) were excluded as data was incomplete at the time of randomization. There were a total of 27,391 callers to the MN FluLine, of which 13,958 reported symptoms of ILI. Of these 3,799 were transferred to a partner NTL. Data was not available from these callers to be able to contact them for this survey. [13].

There was a total of 27,391 calls to the MN FluLine, of which 13,958 (51%) reported symptoms of ILI or being exposed to someone with ILI. Of these 3,691 (26%) were information only/non-flu-related/duplicate calls, 3,799 (27%) were transferred to a partner NTL, and 6,468 (46%) had the nurse protocol administered by the contract NTL of which 374 were prescribed oseltamivir by the NTL [13]. Of the 6,468 calls managed by the contract NTL, 38 calls occurred in March and were ineligible to be included as randomization for the survey occurred in March prior to the completion of MN FluLine operations, leaving 6,430 eligible to be included in the survey (Figure 1).

A total of 1,221 people were called to conduct the evaluation survey including those who completed nurse protocol and those who did not complete nurse protocol groups; 325 agreed to participate for an overall response rate of 26% (Figure 1). Among those who completed the nurse protocol, there were no differences in regards to caller demographics, disposition recommended or oseltamivir prescriptions. Call times to complete the nurse protocol were shorter among persons who responded to the survey compared to persons who did not respond (12.2 vs. 13.3 minutes, p = 0.02). Among those who did not complete the nurse protocol, there were no statistically significant demographic differences between survey responders and non-responders.

309 (27%) callers who completed nurse protocol and 16 (20%) of callers who did not complete the nurse protocol participated in the survey (Table 2). Among those who completed the nurse protocol, 154 (50%) reported an in-person visit with a healthcare professional for their illness, and 58 patients (19%) were prescribed oseltamivir. When asked what they would have done without the MN FluLine, 73% would have sought care (26% would have gone to clinic, 9% to urgent care, 13% to emergency department (ED) and 25% would have called their clinic among those who completed the nurse protocol. Similar responses were observed among those who did not complete the nurse protocol (Table 2).

**Table 2:** Demographics and evaluation survey question responses those who completed the nurse protocol and those that did not complete the nurse protocol.

Survey question	Completed nurse protocol (N = 309)Number (%)	Did not complete nurse protocol (N = 16)Number (%)
**Demographics**		
Median age (range)	23 years (1 month –79 years)	22 years (4 years –7 years)
Female	188 (61%)	10 (63%)
Calling about someone else	170 (55%)	9 (56%)
Reported no health insurance	29 (11%)*	3 (25%)†
**How did you find out about the MN FluLine?**		
Television or radio	84 (27%)	2 (13%)
Healthcare professional	63 (20%)	1 (6%)
Internet	46 (15%)	4 (25%)
Don’t know/don’t remember	36 (12%)	4 (25%)
Family member or friend	30 (10%)	2 (13%)
Employer or information at work	22 (7%)	0
Newspaper	19 (6%)	1 (6%)
Pharmacist or pharmacy	5 (2%)	0
School or daycare	12 (4%)	1 (6%)
Other	12 (4%)	1 (6%)
**Did a nurse attempt to call you back?**		
Yes	–	9 (56%)
No	–	1 (6%)
**If yes, do you remember why you weren’t able to answer the call?**		
Symptoms improved	–	0
Didn’t want to	–	1 (6%)
Symptoms worsened/was too ill	–	1 (6%)
No longer available/bad time	–	2 (13%)
Other	–	5 (31%)
Not reported	–	7 (44%)
**Were you able to follow the nurse’s recommendation?**		
Yes	228 (74%)	–
No	63 (20%)	–
Don’t know/don’t remember	18 (6%)	–
**If not able to follow the recommendation, why not?** §		
Symptoms improved	10 (16%)	–
Symptoms worsened	13 (21%)	–
Didn’t agree with the recommendation	12 (19%)	–
Transportation concerns	1 (2%)	–
Didn’t want to/didn’t have time	2 (3%)	–
No insurance	2 (3%)	–
Insufficient insurance coverage	0	–
Cost issues/concerns	4 (6%)	–
Other	15 (24%)	–
Not reported	4 (6%)	–
**Did you visit a doctor, nurse, or other healthcare professional for your illness?**		
Yes	154 (50%)	8 (50%)
No	140 (45%)	6 (38%)
Don’t know/don’t remember	15 (5%)	2 (13%)
**Did you receive Tamiflu** ‡ **for this illness?**		
Yes	58 (19%)	2 (13%)
No	237 (77%)	12 (75%)
Don’t know/don’t remember	14 (5%)	2 (13%)
**If yes, were you able to take the medication as recommended?** **		
Yes	49 (79%)	2 (100%)
No	8 (13%)	0
Don’t know/don’t remember	3 (5%)	0
No response	2 (3%)	0
**If the MN FluLine had not been available, what would you have done?**		
Gone to clinic	81 (26%)	8 (50%)
Called clinic or provider	77 (25%)	2 (13%)
Gone to emergency department	41 (13%)	1 (6%)
Other	33 (11%)	2 (13%)
Gone to urgent care	28 (9%)	1 (6%)
Done nothing	22 (7%)	1 (6%)
Don’t know/don’t remember	22 (7%)	0
Contact family member/friend	5 (2%)	1 (6%)

* N = 275 as patients were allowed to choose whether to answer this question.

† N = 12 as patients were allowed to choose whether to answer this question.

– Blank indicates that this question was not asked of that survey group.

§ Based on N = 63 who responded they were not able to follow the recommendation.

‡ Trade name for oseltamivir. Trade name was chosen as this was likely more recognizable to survey participants.

** Based on N = 62 for the completed group and N = 2 for the did not complete group who were advised to take oseltamivir (Tamiflu).

**Figure 2:**
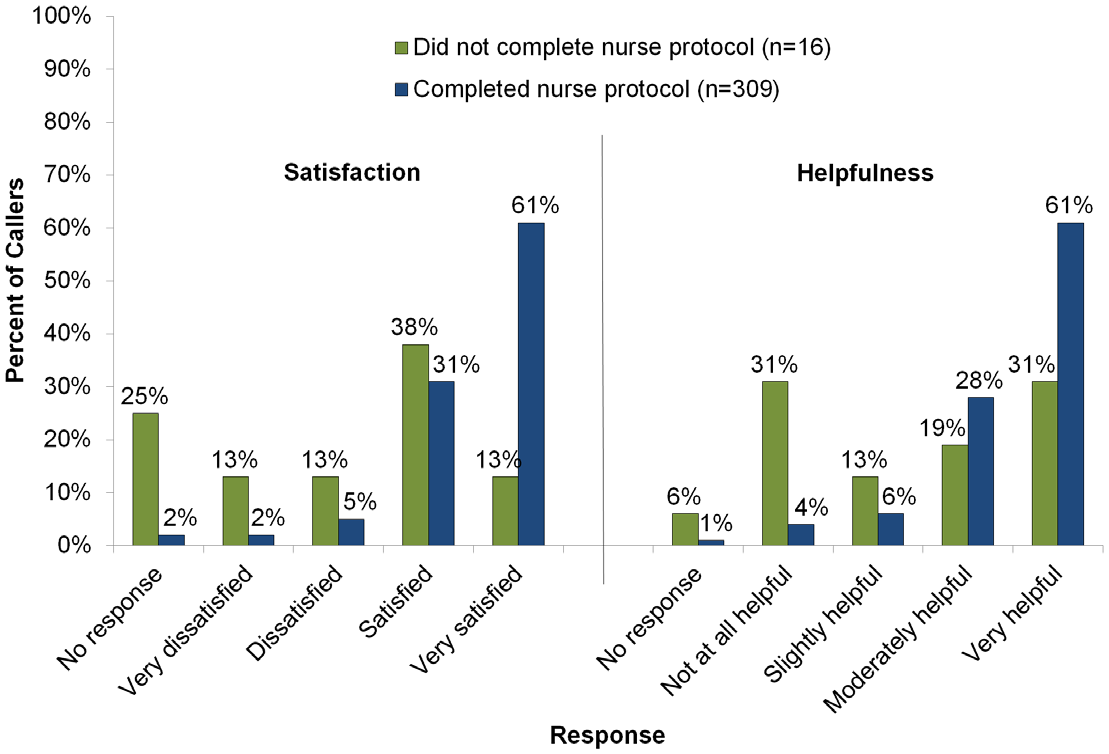
Satisfaction and helpfulness of the MN FluLine among those who completed a nurse protocol and those who did not complete the nurse protocol.

Of those completing the nurse protocol, 274 (89%) reported that the MN FluLine was moderately helpful or very helpful and 282 (91%) were satisfied or very satisfied with their MN FluLine experience (Figure 2). No significant differences were found in reported helpfulness or satisfaction by month of call despite extremely high call volume and longer wait times in the opening weeks of the MN FluLine. No significant differences in reported helpfulness or satisfaction were found between those who were recommended to seek in-person healthcare compared to those who were recommended for home care. Among those who did not complete the nurse protocol, 8 (50%) still found the MN FluLine was moderately helpful or very helpful and 8 (50%) reported being satisfied or very satisfied (Figure 2).

No statistically significant differences in satisfaction or helpfulness of the MN FluLine among those who completed the nurse protocol were found with regard to age of patient, gender of patient, call length or insurance status. In addition, no significant differences were found with regard to visiting a doctor for their illness, receiving oseltamivir, or what they reported they would have done without the MN FluLine. Those who completed the nurse protocol were significantly more likely to report being satisfied or very satisfied compared to those in the group who did not complete the nurse protocol (p<0.001). Similarly, those who completed the nurse protocol felt the service was moderately or very helpful compared to those who did not complete the protocol (p<0.001).

Qualitative data were collected via three open ended questions. A sample of the range in types of statements collected from participants is provided in Table 3. When asked how helpful the MN FluLine was, 78 people provided comments; 57 (73%) were categorized as being positive and included general positive feelings towards the service, appreciating not having to go in to the clinic or ED, and feeling calmed or reassured. Common themes among the 13 (17%) negative comments were not getting the answer they were looking for, too long a wait to get a callback, having H1N1 “hyped” too much by the media, and not having the same person initially answering the phone and administering the triage protocol. Eight (10%) of the comments were classified as neither positive nor negative.

**Table 3:** Sample of qualitative responses categorized as positive, neutral, or negative with respect to the MN FluLine.

How helpful was the MN FluLine to you?		
**Positive comments** (73% of comments)		“It calmed me down immensely. They gave me tips and I followed them.”
		“I knew about the homecare from the news, but it was good to be able to talk to a professional about it.”
		“I think it was a very good service. I hope you do it again next year”
		“Nurse’s recommendation pushed husband to get seen, had collapsed lung hospitalized 11 days”
**Neutral comments** (10% of comments)		“I can’t really remember what they told me”
		“Due to being a nurse, I already knew pretty much what to do at home.”
**Negative comments** (17% of comments)		“I think they thought this [H1N1] was more serious than it really was.”
		“I was seen in the ER before I got a nurse callback.”
**How satisfied were you with your MN FluLine experience?**		
**Positive comments** (72% of comments)	“I was very thankful that these services are out there – would not have been able to afford another visit to the doctor in this economy.”	
	“Glad you were there.”	
	“Very helpful – helped me save money as I have poor insurance. Great service.	
	“A relief to speak to someone without having to leave the house.”	
	“Had blot clots in both lungs - took what nurse said very seriously - really wanted to thank someone”	
**Neutral comments** (11% of comments)	“Had to wait a long time but it was a busy time for everyone”	
	“Got some answers that was helpful but was hoping to get prescription because wife was on chemo”	
**Negative comments** (17% of comments)	“The time it took to get a callback was too long.”	
	“Not helpful, all they told us to do was go in to the doctor – we could have figured that out for ourselves.”	
	“I would have liked to have been able to talk to someone right away when I first called in.”	
**Question: Is there anything else you’d like to add?**		
**Positive comments** (71% of comments)	“It saved me from going in to the clinic and exposing myself to germs. It gave me peace of mind and I’m really glad you were there – thank you!”	
	“It came in handy so I didn’t have to run her in to the hospital in the middle of the night.”	
	“I think it was good to have for people without insurance, like me, to help know when they need to be seen.”	
	"If we have a crisis like that again I hope we have this available."	
**Neutral comments** (16% of comments)	“Spilled previous Rx for Tamiflu* and needed a refill because she could not afford to take the child back to urgent care.”	
	Caller would have liked the state to test more people with H1N1 symptoms. They wanted to know and received no help.	
**Negative comments** (13% of comments)	“I would have liked nurses to have more advice and information on high fevers - would have saved a trip to the ER.”	
	“Would have been nice to get a callback sooner.”	

* Trade name for oseltamivir.

When asked about their satisfaction with the MN FluLine, 181 people provided comments. Of these comments, 131 were positive (72%), 19 neutral (11%) and 31 were negative (17%). Common themes among positive comments were positive feedback about the service, receiving information that was helpful, and thankfulness at not having to go in to the ED or clinic. Among negative comments, common themes were frustration over the lag in callback time, receiving information that was not helpful, and not agreeing with the information given.

Finally, participants were asked if there was anything else they’d like to comment on and 134 callers provided comments of which 95 (71%) were positive, 21 (16%) neutral, and 18 (13%) negative. Common themes for positive comments included general positive feedback, information received was good and useful, and nurses being helpful. Common themes from negative comments included receiving information that was perceived to be wrong or not helpful and complaints over waiting too long for a callback.

The contract cost paid by MDH for the operation of the MN FluLine was $331,226. This cost included procurement and operations of the toll-free phone number and associated infrastructure needs. This also included transferring callers to their healthcare system nurse triage line if they had insurance and providing nurse triage services to those who did not have insurance or their healthcare system did not have a nurse triage line. The 27,391 calls were managed with an average cost of $12.09 per call.

## Discussion

The MN FluLine was a statewide NTL intervention implemented to respond to the public health threat of 2009 H1N1 influenza and included prescribing of antivirals. Investing in a toll-free number and agreeing to operate in a coordinated fashion, MDH and the healthcare systems in Minnesota with nurse triage lines were able to augment the current healthcare system at a time when it was highly strained. The inclusion of prescribing oseltamivir allowed callers to be served without the need for an in-person healthcare visit if antiviral treatment was indicated. This service had an immense impact on the MN population and healthcare community reaching over 27,000 callers in need of services [13]. The program evaluation based on a random sample of callers had an overall response rate of 26%, similar to findings from other evaluation studies conducted among nurse triage line callers that had 16% and 25% response rates [14], [15]. Costs associated with utilizing the MN FluLine were estimated at slightly more than $12 for each call. This is noteworthy, especially when compared with estimates of in-person healthcare delivery in the US Midwest (typically not including medication costs) at $876 per emergency department visit, $269 per urgent care visit, and $192 per clinic visit for upper respiratory infection and fever visit [16].

For callers with ILI who received the full MN FluLine intervention (i.e. completed nurse protocol), the vast majority reported that they were either very satisfied or satisfied and felt the service was very or moderately helpful. Although the numbers are small, and may not be representative, this is similar to findings regarding satisfaction from other telephone triage services [17], [18]. It was surprising to find that half of those who did not complete the nurse protocol reported being very satisfied or satisfied since this group did not receive the full intervention as it was intended, but it is possible that callers felt the existence of the service was valuable and satisfactory even if they themselves did not fully benefit from it. Qualitative comments provided by callers were very useful in providing clarity in areas that were successful and for learning about ways to improve the service during future applications.

The program evaluation identified several key aspects for improvement if a similar nurse triage line will be utilized in the future. First, the need was substantially underestimated and planning for far greater phone line and personnel resources initially which could be scaled back, would have improved the service. Second, callers expected to be connected to a nurse right away and greater public education about the process (i.e. talking to a medical screener first and then a nurse) would have improved this experience. Third, advance development of triage protocols instead of creating them *de novo* would have decreased the development time. Finally, advance planning with healthcare system, including healthcare plans, and healthcare delivery organizations and pharmacy representatives would have allowed for a more rapid implementation.

Fundamentally the MN FluLine was designed and executed rapidly to meet a major public health need. Had it been designed as a pilot project, it would have been useful to collect additional data that would have enabled additional assessment including a thorough cost-analysis. The evaluation survey was conducted several months after the launch of the MN FluLine creating the possibility for recall bias. We did not, however, see a difference in satisfaction or helpfulness by month of call which would have suggested recall bias. We kept the survey brief to increase participation, but this also meant we were limited in the data we were able to collect from callers. We utilized a cross-sectional post-test-only study design which does not allow us to factor in baseline measures. We did, however, utilize a random sample of callers to minimize sampling bias. Evaluation data were based on self-report. Since callers reported their survey responses to a nurse there is the potential for social desirability bias in their responses. Finally, wide implementation of NTLs may be limited by state and local laws. In MN, prescribing by a nurse protocol under physician supervision is allowed but this may not occur elsewhere. An assessment of federal and state law could provide additional insight into potential limitations to broad implementation.

Phone triage lines represent a powerful tool for public health. It provides the opportunity for flexible implementation of healthcare delivery during an emergency and a portal to consistent information. Creating a system to allow for early assessment and intervention likely prevented influenza-associated morbidity and possibly additional influenza illnesses. Further, it provides a system of clinical evaluation that is accessible in places where access to the internet or distance may limit rapid deployment of healthcare services. Such a service is likely to be cost savings to our entire healthcare system and allows the leveraging of existing call center infrastructure. Under the current United States healthcare system the financial benefits provided by such a system are not easily quantified due to the fractionated healthcare delivery and the fiscal division of the agents that bear the costs from those that receive benefits. The private-public partnership shared the cost (financial, infrastructure, and personnel). Federal, state, and local planners should consider phone triage lines as an important tool in response to future emergency response.
